# Mitochondrial Dysfunction and Cellular Senescence Due to Aging Contributes to Prostatic Fibrosis

**DOI:** 10.1093/geroni/igaa057.439

**Published:** 2020-12-16

**Authors:** Teresa Liu, Emily Ricke, Donald DeFranco, William Ricke

**Affiliations:** 1 University of Wisconsin--Madison, Madison, Wisconsin, United States; 2 University of Wisconsin - Madison, Madison, Wisconsin, United States; 3 University of Pittsburgh, Pittsburgh, Pennsylvania, United States; 4 University of Wisconsin Madison, Madison, Wisconsin, United States

## Abstract

Aging is the single largest risk factor for many common diseases that burden public health. This is especially true in the prostate; as men age, the prostate undergoes a prototypical aging change, fibrosis. The aged fibrotic prostate causes urinary symptoms which will afflict nearly every man if they live long enough. However, the molecular mechanisms responsible for the aging-dependent promotion of fibrosis are largely unknown and understudied. In this study, we sought to reveal the contribution of mitochondrial dysfunction to fibrosis in the aging prostate, ultimately leading to prostate dysfunction, urinary symptoms, and overall poor health. The demise of mitochondrial function is well established in other aging-associated diseases but has not been investigated in the prostate. Our analysis revealed an increase in cellular senescence and mitochondrial dysfunction in BPH patient tissue compared to normal prostates. Furthermore, selective inhibition of mitochondrial complex I by rotenone in cultured human prostate fibroblasts led to myofibroblast phenoconversion as characterized by increased expression of Col1a1, Col3a1, and ACTA2. To determine the contribution of mitochondrial dysfunction on fibrosis and lower urinary tract dysfunction (LUTD), we examined the prostate lobes in both aging and steroid-induced LUTD in mice. These models have been extensively characterized and exhibit an age-mediated increase in LUTD and fibrosis. We observed a decrease in mitochondrial function and an increase in cellular senescence corresponding to an increase in fibrosis and LUTD. This suggests that normal aging-dependent reductions in mitochondrial complex I function in the prostate may promote fibrosis and contribute to urinary dysfunction.

